# Classroom assignments as measures of teaching quality

**DOI:** 10.1016/j.learninstruc.2017.08.001

**Published:** 2018-04

**Authors:** Jeanette Joyce, Drew H. Gitomer, Charles J. Iaconangelo

**Affiliations:** Rutgers University Graduate School of Education, 10 Seminary Place, New Brunswick, NJ 08901, United States

**Keywords:** Assignments, Teaching quality, Intellectual demand

## Abstract

We investigate classroom assignments and resulting student work to identify important characteristics of assignments in terms of instructional quality and their validity as measures of teaching quality. We examine assignment quality within a large-scale project exploring multiple measures including classroom observations, teacher knowledge measures, and value-added estimates based on student achievement scores. Analyses included descriptive statistics, multivariate analyses to understand factors contributing to score variance, and correlational analyses exploring the relationship of assignment scores to other measures. Results indicate relatively low demand levels in all teacher assignments, a marked difference in score distributions for mathematics (math) and English language arts (ELA), and a substantial relationship between what was asked of and produced by students. Relationships between assignments scores, classroom characteristics, and other measures of teaching quality are examined for both domains. These findings help us understand the nature of and factors associated with assignment quality in terms of intellectual demand.

## Highlights

• Intellectual demand of assignments for math and English language arts is limited.
• Intellectual quality of student work is systematically related to assignment demand.
• Assignment quality is related to classroom demographics and academic achievement.
• Assignments complement classroom observations as a measure of classroom interaction.

## 1 Introduction

### 1.1 Rationale

Central to recent educational accountability efforts are teacher evaluation systems that include measures of the quality of classroom interactions, with the underlying claim that what teachers do in the classroom matters (Stodolsky, 1990). Classroom observations have received the greatest amount of attention in evaluating classroom interactions (e.g., Bell et al., 2012, Gitomer et al., 2014, Kane et al., 2014, Taylor and Tyler, 2012). They provide important evidence about classroom interactions and guide interpretation of classroom interactions to make inferences about an array of classroom qualities including the goals that teachers have for students, the depth of content and reasoning that characterizes a given lesson, and classroom discourse. Formal teacher evaluation, predominantly focused on classroom observations, characterizes most OECD countries (OECD, 2013a, Scheerens et al., 2012). Across OECD countries, the overarching articulated goal of evaluation is to improve teaching quality (OECD, 2013b).

Within the context of teacher evaluation systems, very little attention has been given to classroom artifacts as a direct source of evidence about the quality of classroom instruction. Yet, students spend a great deal of their instructional time working on and with assignments, whether they are instructional tasks or some type of assessment. Artifacts have been used as part of larger portfolios of teaching (e.g., OECD, 2013b, Stake et al., 2004) but not as standalone evidence of teaching quality.

The current study examines the quality of assignments in middle school mathematics (math) and English language arts (ELA) classrooms as part of a larger study of measures of teaching quality. We define teaching quality as “the quality of interactions between students and teachers; while teacher quality refers to the quality of those aspects of interactions that can be attributed to the teacher” (Bell et al., 2012, pp. 63–64). We acknowledge that, in many cases, assignments may not simply reflect instructional decisions of the teacher. Assignments may be part of a curriculum that is determined at the school or district level. Thus, assignments can also provide information about what the district holds as its view of quality teaching, with the teacher acting as “a key connection between policy and practice …” (Cohen & Hill, 2000, p. 329).

This study is intended to provide evidence that classroom assignments through collected artifacts can provide complementary interpretations about classroom interaction quality. We investigate how a protocol can be used to assess the quality of teaching practice and student learning by evaluating the quality of assigned quizzes, tests, and in-class work. These artifacts are, in certain ways, more straightforward to interpret than are classroom observations. Specifically, assignment artifacts can make clear what is expected of students and how students respond to those expectations in ways that are not always observable within a set of classroom interactions (see Gitomer & Bell, 2013). This study also contributes further validity evidence (see Kane, 2013) for the interpretation of scores from an artifact protocol.

As part of a larger study of a broad set of measures of teaching quality, this study investigates the relationship of artifact scores to measures of classroom observations, teacher knowledge, and value-added measures based on standardized student achievement tests. Acknowledging that “no single measurement can capture the full range of teacher performance in different contexts or conditions” (Looney, 2011, p. 443), this research enables us to consider what artifacts can contribute to an understanding of teaching quality and how scores on artifacts are related to scores on other teaching quality measures.

As with classroom observations, *quality* of classroom assignments is a construct that has no absolute definition. Therefore, a given protocol provides a conceptual lens through which quality is defined (e.g., see Dwyer, 1998). For this work, we adopt the framework of authentic intellectual work (Newmann, Bryk, & Nagaoka, 2001). Originally proposed by Archibald and Newmann (1988), the framework characterizes the work students are typically asked to do as contrived and superficial and contrasts that with the kinds of work skilled adults often do. Authentic intellectual work is viewed as relatively complex and socially or personally meaningful.

Newmann et al. (2001) describe authentic intellectual work as having three distinctive characteristics. First, it involves the *construction of knowledge,* arguing that authentic work requires one to go beyond routine use of information and skills previously learned. Problem solvers must construct knowledge that involves “organizing, interpreting, evaluating, or synthesizing prior knowledge to solve new problems (p. 14).” The second characteristic of authentic intellectual work is *disciplined inquiry,* which involves the use of prior knowledge in a field, in-depth understanding, and elaborated communication. The final characterizing feature is *value beyond school*, the idea that work that people do authentically is intended to impact or influence others.

The principles of authentic work derive from philosophies and studies from constructivist traditions including Bruer, 1993, Dewey, 1916, Resnick, 1987, and Wolf, Bixby, Glenn, & Gardner (1991). Support for these constructivist pedagogies, on which the authentic work framework is based, include Carpenter et al., 1989, Cobb et al., 1991, and Silver and Lane (1995). In this constructivist tradition, students engage with real-world problems that have legitimacy within their own experiences and that involve the structuring and restructuring of knowledge rather than simply reporting back information that they have reviewed.

A number of studies have provided empirical support. Newmann, Marks, and Gamoran (1996) studied a set of restructured schools that were designed around authentic intellectual engagement and related constructivist practices. They found that authentic pedagogical practice explained approximately 35% of the variance in student performance. D'Agostino (1996) studied compensatory (low-achieving) education third-grade classrooms and found a strong relationship between authentic instruction and math problem solving. Findings for reading comprehension were more ambiguous. Knapp, Shields, and Turnbull (1992) studied high-poverty schools and found that those classrooms that engaged in authentic practices of meaning making, disciplinary thinking, and connections with the real world produced students who were substantially stronger in their academic attainment.

The framework of authentic intellectual engagement is the foundation of the artifact protocol used in this study, the *Intellectual Demand Assignment Protocol (IDAP)* (Wenzel, Nagaoka, Morris, Billings, & Fendt, 2002). While other assignment protocols build on different frameworks, the assignment protocols cited in the literature all focus on some variation of intellectual demand.

Prior classroom assignment research has provided understanding of the intellectual demands that are placed on students, how students respond, and how these assignments affect student outcomes. Students respond to authentic work that is challenging, constructive, and relevant (American Institutes for Research, 2003, Beane, 2004, Daggett, 2005, Dowden and Relevant, 2007, Milanowski et al., 2009, Ng, 2007, Paik, 2015, Prosser, 2006, Woolley et al., 2013). Intellectual demand has also been measured reliably (Borko et al., 2005, Clare and Aschbacher, 2001, Matsumura et al., 2008) and is connected to student outcomes (Matsumura and Pascal, 2003, Mitchell et al., 2005, Newmann et al., 2001).

This study is situated within a larger validation effort of measures of teaching quality. Following Messick (1989) and Kane (2013), we investigate evidence of the extent to which scores from an artifact protocol support the appropriateness of inferences about teaching quality in middle school math and ELA classrooms. Adopting a theoretical framework of intellectual demand, this research seeks empirical support for the use of artifacts to make judgments of teaching quality by investigating the following questions:

1. How are scores representing assignment intellectual demand distributed for math and ELA?
2. What is the relationship between the intellectual demand of a given assignment and the student work produced in response?
3. What are the relationships between assignment intellectual demand and other measures of teaching?
4. How are assignment scores related to contextual variables including teacher characteristics, class demographics, schools, or districts?

### 1.2 Review of research on assignment quality as measures of classroom practice

Initial validation work of IDAP (Wenzel et al., 2002) provided evidence that IDAP scores could support inferences about the quality of classroom assignments in the Chicago Public Schools. Artifacts could be rated reliably, though there was some year-to-year drift. In addition, more challenging artifacts were associated with higher test scores (Newmann et al., 2001). Note, however, these initial validation studies used status scores rather than a value-added measure. They also did not include observation measures as alternative measures. Additional work supporting the validity of using IDAP was done by Mitchell et al. (2005), who also demonstrated that artifacts could be scored reliably and that scores were related to status achievement scores.

Studies using other assignment protocols have also examined the validity and reliability of classroom assignments. Matsumura and colleagues found that a reliable estimate of ELA classroom assignment quality could be attained with three assignments, that there was overlap among the scales, and that there was a relationship between assignment quality and other measures of teaching quality (Clare, 2000, Clare and Aschbacher, 2001, Clare et al., 2001, Matsumura and Pascal, 2003, Matsumura et al., 2008). Similar work looking at middle school mathematics and science classes found that a reliable estimate of classroom practice could be based on teacher assignments and student work (Borko et al., 2007, Borko et al., 2005).

Studies have also explored factors related to the quality of assignments in a variety of contexts. Koh and Luke (2009) also found a relationship between quality of teacher assignments and student work in Singapore middle school social studies, science, English, and math classrooms. Students of different cultural and socioeconomic backgrounds receive lower-quality assignments, even in “progressive” settings (Auerbach, 2002, Rubin, 2003), suggesting that access to intellectually demanding assignments is not uniform across classrooms and schools.

### 1.3 Assumptions underlying the IDAP protocol

Every protocol, including IDAP, makes a set of assumptions about its range of application and the specifics of how evidence is to be collected and assessed (Wenzel et al., 2002). Several key assumptions described below guided the design of this study.

#### 1.3.1 Omission of the artifact origin

In considering demand, the focus is on what students are asked to do, independent of who actually designed the artifact. This study is not an evaluation of a teacher's ability to design instructional artifacts, including assessments, nor should scores be influenced by whether the artifact was authored by a commercial publisher, a school district, or the teacher herself.

#### 1.3.2 Differentiation of challenging and typical assignments

Teachers assign particular work to satisfy different educational purposes, and some artifacts will not be as intellectually challenging as others. In some classes, for example, teachers may assign a relatively routine worksheet to develop a set of procedural skills. In another class, the same teacher might assign a complex project that requires extended disciplinary inquiry and communication. Thus, this study followed Wenzel et al.’s model (2002) and collected both challenging and typical assignments as part of IDAP.

#### 1.3.3 Treatment of subject areas

While the *construct* of intellectual demand cuts across all subject areas (Newmann, King, & Carmichael, 2007), the *manifestation* of intellectual demand is domain-specific. Construction of knowledge, disciplinary inquiry, and the audiences and purpose have meaning specific to respective disciplines. Thus, the study, following Wenzel et al. (2002), includes separate protocols for math and ELA, all within the common framework of authentic intellectual demand.

#### 1.3.4 Treatment of grade level

As with subject areas, the framework of authentic intellectual demand is deemed to be appropriate across grade levels (Newmann et al., 2007). The construct of intellectual demand is not based on the grade-associated difficulty of the content expressed in the task but on the depth of the thinking about the content expected of the student.

#### 1.3.5 Treatment of practices

IDAP does not privilege particular teaching practices. Newmann et al. (2007) argue that “no single practice or set of practices has been shown to be most effective for varied intellectual outcomes for most students across several grade levels and subjects” (p. 15).

#### 1.3.6 Selection of artifacts

This research develops from, but differs from, earlier work done using Teacher Work Samples (Denner, Salzman, & Bangert, 2001) by not eliciting specific artifacts, but instead allowing for teacher decision-making (Dwyer, 1998) in selecting artifacts to share, consistent with Wenzel et al. (2002).

## 2 Method

### 2.1 Sample

The study, conducted across two years, included three school districts within a large metropolitan area in the United States, across 47 middle schools and with 225 math and 225 ELA teachers who volunteered to participate. Teachers were asked to supply six assignments across the school year. Per the IDAP protocol (Wenzel et al., 2002), we asked teachers to provide both typical assignments and those that they considered to be challenging. Teachers had latitude in determining what constituted “typical” and “challenging.” While a *typical* assignment was described to teachers as “everyday work,” a *challenging* one was described as “an assignment that gives you the best sense of how well your students are learning a subject or skill at their highest level.” No distinctions were made as to whether the assignments were produced by the teacher, a commercial publisher, the school district, or any other entity.

**Fig. 1:**
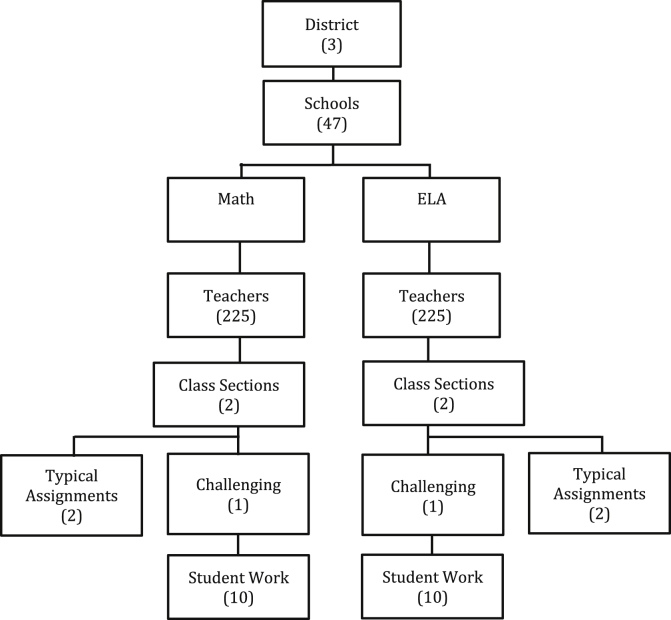
Study data structure.

The structure of the data is summarized in Fig. 1. Two classrooms (*class sections*) of each teacher were sampled. In the fall, a teacher selected a typical assignment from each of her two different class sections and one sample of a challenging assignment from one of the class sections. In addition, per Wenzel et al. (2002), p. 10 samples of student work associated with each challenging assignment were randomly selected from the classroom (in some cases fewer than 10 were available). This procedure was repeated in the spring, except that the challenging assignment came from the other class section. Half the teachers were sampled each year of data collection, yielding a total of 904 typical and 451^1^ challenging assignments for each subject area. There were 4396 student work artifacts for math and 4390 for ELA.

### 2.2 Instruments

The study used the IDAP (Wenzel et al., 2002) to examine classroom assignments and associated student work within a framework that valued authentic intellectual engagement. Two separate protocols, one for math and one for ELA, were used in the current study. For math, intellectual demand was considered in terms of three dimensions: communication, conceptual understanding, and real-world connections. For ELA, intellectual demand dimensions included: communication, construction of knowledge, and real-world connections. Teacher assignments and student work were each evaluated separately, and the scales for each were similar but not identical. The structure of the scales for assignments and student work is presented in Table 1. The rubrics for each of the respective scales are available in Appendix A.

**Table 1:** Scale dimensions (scale range) for teacher assignments and student work in math and ELA.

Scale	Teacher Assignment (Math)	Student Work (Math)	Teacher Assignment (ELA)	Student Work (ELA)
1	Written Communication (1–3)	Written Communication (1–3)	Elaborated Communication (1–4)	Elaborated Communication (1–4)
2	Conceptual Understanding (1–4)	Conceptual Understanding (1–4)	Construction of Knowledge (1–3)	Construction of Knowledge (1–3)
3	Relevant Context/Real-World Connection (1–4)	Reasoning (1–4)	Real-World Connection (1–4)	Language Conventions (1–4)

#### 2.2.1 Raters, training, and scoring

Artifacts were scored within domains by raters who had expertise both in teaching math (n = 17) or ELA (n = 22) and in using scoring rubrics to score constructed responses for other standardized assessment programs. Five highly experienced individuals in constructed-response scoring served as scoring leaders for each domain, providing guidance and oversight throughout the scoring process. Artifacts were distributed and displayed to raters using a proprietary scoring tool. Exemplar selection and rater training were led by experts (one each for math and ELA), who were involved in the original development of the IDAP. Papers were chosen by the experts from the pool of artifacts submitted as part of the study to serve as exemplars for each rubric and each score point.

Training and certification followed the same process for each of the six rubrics in each domain. First, benchmark papers were introduced to illustrate each score point. Then, using a set of 6 training papers, raters first scored the artifact, followed by a discussion with the scoring trainer. Next, each rater independently completed a set of 6 certification papers and was required to assign the pre-determined score in at least four cases (with no deviations of more than 1 score point) in order to begin the actual scoring. Training took between 2.5 and 5 h per scale. During scoring of the assignments, typical and challenging assignments were intermixed and rated in the same session. For student work, only challenging assignments were involved. Scoring leaders regularly read scored samples to ensure reliability. Scoring of the entire sample on each scale took 1–3 days, depending on the complexity of the scale and the number of artifacts.

### 2.3 Other measures of teaching quality

In addition to the IDAP, the study included other measures associated with teaching quality.

#### 2.3.1 Observation protocols

Each teacher in this study was observed four times, and each lesson was video-recorded. Three different protocols were used to score each of the four videos. All teachers were scored using the Framework for Teaching (Danielson, 2013) and CLASS™ (La Paro & Pianta, 2003). These are protocols designed to capture qualities of instruction, classroom environment, and interpersonal relationships across all subjects and grades. In addition, math teachers were scored using the subject-specific Mathematical Quality of Instruction (MQI) protocol (Learning Mathematics for Teaching Project, 2011), and ELA teachers were scored using the subject-specific Protocol for Language Arts Teaching Observation (PLATO) (Grossman et al., 2009). Additional details on the design, rater training, and collection of the observation protocol data are provided in Bell et al. (2012). The observation lessons were collected independently from the assignments, meaning that the foci of the two sources of evidence were not linked in terms of specific content being studied. The estimated reliability of the observation scores at the teacher level using four observations was approximately 0.65 (Bill & Melinda Gates Foundation, 2012). Reliability was estimated using a variance decomposition analysis that included teacher, section, lesson, rater, and residual factors.

#### 2.3.2 Value-added modeling

VAM scores were computed at the teacher by class section level, using state standardized achievement tests. The dataset also included scores from the state accountability tests in math, ELA, reading, and science, longitudinally linked to individual students across grades. Finally, the data included student demographic and program participation information including race, gender, free or reduced price lunch status, English language learner (ELL) status, and indicators of participation in gifted or special education programs. VAM scores were computed using the latent regression model (Lockwood & McCaffrey, 2014), which regresses outcome test scores on teacher indicator variables, student background characteristics, and student prior test scores while accounting for the measurement error in the prior test scores. Reliabilities for VAM scores using a variance decomposition analysis were 0.89 for math and 0.80 for ELA (Lockwood, Savitsky, & McCaffrey, 2015).

#### 2.3.3 Teacher knowledge

All math teachers took a version of the Mathematics Knowledge for Teaching (MKT) test (Hill, Schilling, & Ball, 2004). The 38-item instrument was designed to assess “the mathematics teachers use in classrooms” (Delaney, Ball, Hill, Schilling, & Zopf, 2008, p. 172) through items that ask questions covering instructional choices and analysis of student answers in the areas of number, operations, and content knowledge as well as patterns, functions, and algebra content knowledge. For ELA, teachers were administered an abridged form (30 items) of the Praxis^®^ test used for ELA teacher certification. This instrument is intended to assess “ … knowledge, skills, and abilities believed necessary for competent professional practice” (Educational Testing Service, 2015, p. 5). Reliabilities for teacher knowledge scores were 0.85 for math and 0.78 for ELA (Lockwood et al., 2015).

## 3 Results

### 3.1 How are scores representing assignment intellectual demand distributed for math and ELA?

To determine scale scores we first calculated an average score of all ratings for a given artifact on a given scale. We then averaged those scale scores to calculate a total assignment score.

Initially, however, it is important to establish that any variation in assignment scores is not a function of an undesirable confound, such as raters. In order to understand factors that might influence assignment scores, a variance component analysis was conducted. The assignment scale ratings were modeled via a multi-level cumulative logit mixed model (see Appendix B for details). Fitting this model allowed for a look at the reliability of the raw scores by computing the proportion of modeled variance accounted for by the school, teacher, classroom, assignment, and rater (see Table 2). Raters are associated with only 3% of the modeled variance for both math and ELA. In contrast, assignments account for 72% and 95% of the modeled variance for math and ELA, respectively. This suggests that most of the raw scale score variation is due to differences in assignment quality rather than any rater effects.

**Table 2:** Variance components analysis for total assignment quality scores.

Component	Math Variance (%)	ELA Variance (%)
School	0.07 (6)	0.02 (1)
Teacher	0.04 (4)	0.03 (1)
Class Section	0.17 (15)	0.00 (0)
Assignment	0.81 (72)	2.39 (95)
Rater	0.03 (3)	0.08 (3)

To account for any potential residual rater effects as well as the possibility that some scales are more stringent than others (i.e. it is more difficult to obtain a high score on a particular rubric), a modeling approach was used to estimate true assignment scores. Linacre’s (2015) Many-Faceted Rasch Model (MFRM), the same IRT modeling approach used in Newmann et al. (2001) and Mitchell et al. (2005), was used to estimate assignment intellectual demand^2^ (for further information, see http://www.winsteps.com/facetman/theory.htm).

Modeling did not have a substantial effect on the study results. The relative ranking and distribution of assignment scores is highly consistent between the MFRM adjusted overall scores and the simple averages computed across scales (see Table 3). Correlations between adjusted and raw averages range from 0.97 to 0.99 across all scales. The modeled or “adjusted” scores are used in all further analyses that include overall assignment quality scores, and raw scores are used in analyses focused on specific scales.

**Table 3:** Teacher assignment raw and adjusted scores (SD).

	Math Raw	Math Adjusted	ELA Raw	ELA Adjusted
Typical	1.73 (0.41)	1.75 (0.42)	1.61 (0.61)	1.58 (0.59)
Challenging	1.89 (0.49)	1.90 (0.50)	2.03 (0.71)	2.05 (0.74)

*Note.* Range = 1–3.67. *df* - Typical = 904; *df* - Challenging = 451.

#### 3.1.1 Math

**Fig. 2:**
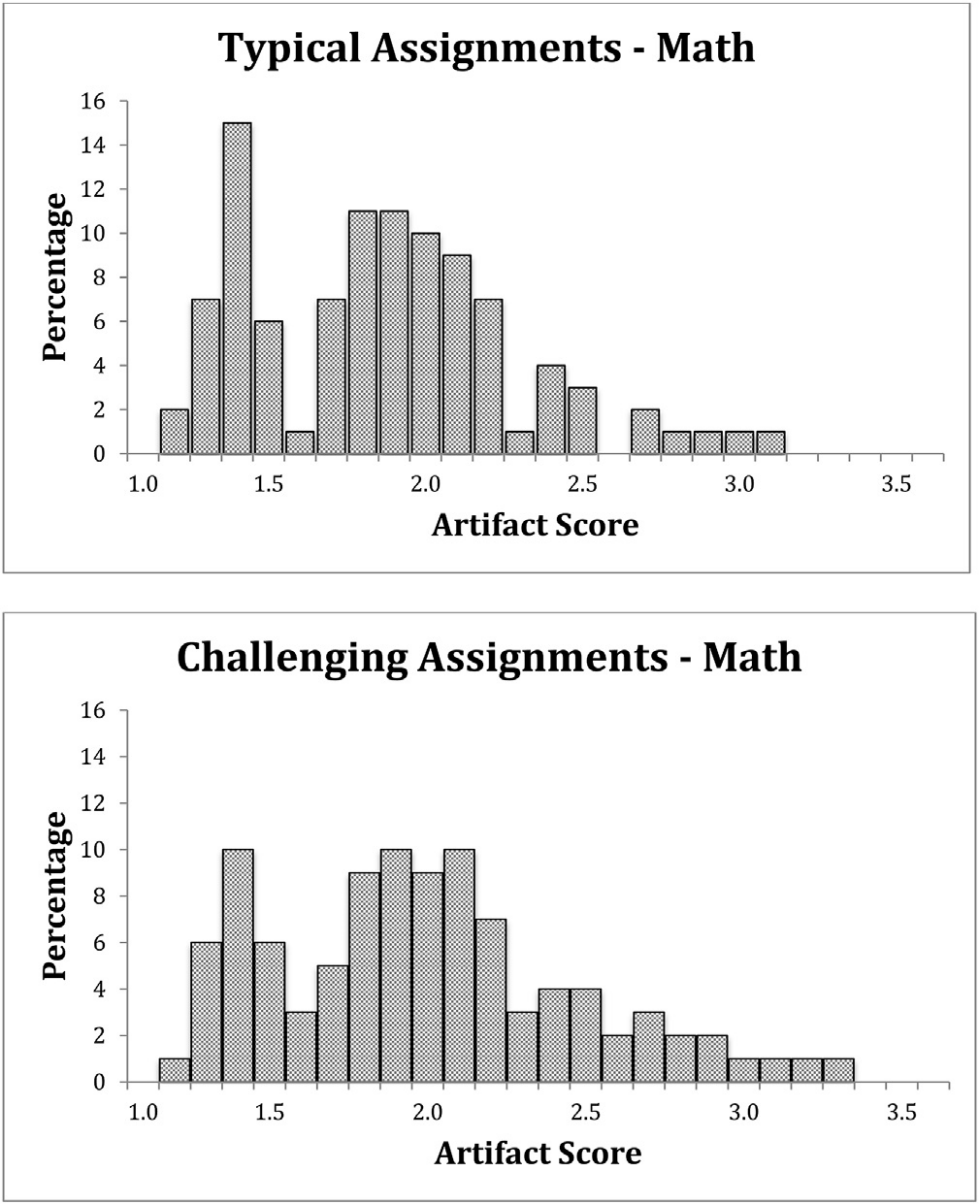
Math assignment score distributions.

Average intellectual demand scores are relatively low with respect to the overall scale (see Table 3). Fig. 2 shows a large proportion of scores below 2 on a scale that ranges from 1 to 3.67 for both typical and challenging assignments. Though the challenging assignments show higher intellectual demand, they are still relatively lacking in demand for substantial demonstration of conceptual understanding, explanation of solution path, or connection to real-world issues. Differences between typical and challenging scores indicate that there is an increase in demand though the overall distribution of scores remains highly restricted. A paired *t*-test comparing the average adjusted scores of typical and challenging assignments from the same teacher indicates a small yet statistically significant difference between the two types of assignments (*t* (443) = 6.1, *p* < 0.001, *d* = 0.16). Given the skewness of the data, however, the precise estimate of any effect sizes should be interpreted with appropriate caution.

#### 3.1.2 ELA

**Fig. 3:**
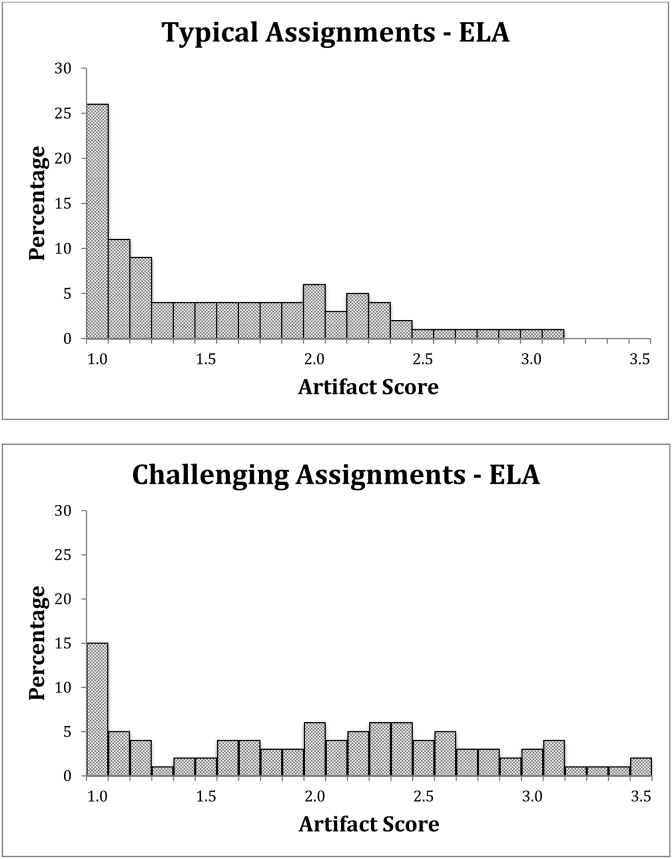
English language arts (ELA) assignment score distributions.

There is a relatively low level of intellectual demand for typical assignments, with a mean of 1.58 on a scale that extends to 3.67. Most assignments received a score of less than 1.5. There is a clear difference in the distribution of challenging scores as many more assignments received scores of 2 or higher (see Table 3 and Fig. 3). Although data for challenging assignments is still skewed, it is also more evenly distributed across the scale. Typical and challenging ELA assignment adjusted scores from the same section are also statistically different (*t* (444) = 12.7, *p* < 0.001, *d* = 0.48).

#### 3.1.3 Performance on individual scales

Table 4 presents information on each of the scales contributing to the overall intellectual demand score for both typical and challenging assignments in math and ELA. Differences between the typical and challenging assignments are also statistically significant at the scale level in both domains.

**Table 4:** Mean assignment demand by scale (SD)(n) including differences between typical and challenging scales.

Scale	Math Typical	Mean Difference^a^	Math Challenging	Scale	ELA Typical	Mean Difference^a^	ELA Challenging
Written Communication (3-point scale)	1.52 (0.60) (900)	0.21^∗∗∗^ *ES* = 0.35	1.73 (0.68) (448)	Elaborated Communication (4-point scale)	2.01 (0.96) (895)	0.66^∗∗∗^ *ES* = 0.69	2.66 (1.08) (443)
Conceptual Understanding (4-point scale)	2.19 (0.44) (901)	0.10^∗∗∗^ *ES* = 0.23	2.29 (0.54) (447)	Construction of Knowledge (3-point scale)	1.57 (0.69) (899)	0.43 *ES* = 0.62	1.99 (0.77) (444)
Real-World Connection (4-point scale)	1.50 (0.63) (901)	0.13^∗∗∗^ *ES* = 0.21	1.61 (0.70) (446)	Real-World Connection (4-point scale)	1.27 (0.53) (899)	0.22 *ES* = 0.42	1.49 (0.64) (448)

*Note.* For this analysis the *n* reflects total number of ratings contributing to the scale, including cases with two ratings.

*p < 0.05, **p < 0.01, ***p < 0.001.

a ES stands for effect size.

For math, all scales scores are slightly higher for challenging than for typical assignments. More challenging assignments are somewhat more likely to ask students to show their work or provide some types of explanation. The other scales have smaller differences for assignment type, although all score differences between challenging and typical assignments are statistically significant.

For ELA, score differences between typical and challenging assignments are larger. Challenging assignments are more likely to ask for some extended writing as a characteristic associated with rigor and construction of knowledge and more frequently ask students to take on a role in their writing. However, very rarely are these roles judged to be real world for the student (as described by the lack of scores of 3 and 4 on the relevance scale).

Table 5a and Table 5b present the correlations among scales for math and ELA, respectively. After aggregating scale scores at the teacher level and pooling across all assignments, the correlations were computed using Spearman's rank correlation Rho. The correlation reported in each series pools scores across all assignments. Analyses that included only typical or only challenging assignments yielded similar results. All correlations are statistically significant, yet moderate, supporting the idea that the IDAP dimensions are each measuring related yet unique constructs.

**Table 5a:** Assignment inter-scale correlations for mathematics.

Scale	Written Communication	Conceptual Understanding	Real-World Connection
Written Communication	1		
Conceptual Understanding	0.52	1	
Real-World Connection	0.38	0.33	1

**Table 5b:** Assignment inter-scale correlations for English language arts (ELA).

Scale	Elaborated Communication	Construction of Knowledge	Real-World Connection
Elaborated Communication	1		
Construction of Knowledge	0.75	1	
Real-World Connection	0.46	0.52	1

*Note.* All correlations *p* < 0.01.

### 3.2 What is the relationship between assignment quality and resulting student work in math and ELA?

This analysis examines the relationship between the demands contained in classroom assignments and the corresponding work that students do to fulfill the assignment. Student work scores were regressed onto the teacher assignment ratings using hierarchical linear modeling (HLM; Raudenbush & Bryk, 2002) to describe the overall relationship of teacher assignment quality and quality of student work.

To better understand this relationship, we divided each scale into thirds and categorized each assignment as low, medium, or high and then reported the mean student work score for each category (see Table 6).

**Table 6:** Mean student work score by level of assignment demand (SD) (n)d.

Demand Level of Teacher Assignment	Math	ELA
All	1.37 (0.40) (4396)	2.04 (0.90) (4390)
Low (<1.89)	1.26 (0.28) (2370)	1.38 (0.59) (1781)
Medium (1.89 =<>= 2.78)	1.46 (0.44) (1764)	2.37 (0.74) (1809)
High (>2.78)	1.70 (0.63) (262)	2.78 (0.59) (800)

#### 3.2.1 Math

For math, more than 2000 student artifacts were produced in response to low-demand assignments and were uniformly rated low in demand. As intellectual demand of the assignment increased, there is a slight increase in quality of associated student work, but student scores remain relatively low. The weak relationship was confirmed by regressing student work quality onto teacher assignment quality ratings with a HLM, which returned a standardized coefficient of only 0.23 (*t* = 15.5), with an *R*^*2*^ value of only 0.14. That is, math assignment ratings accounted for only 14% of the observed variance in student work scores. The most salient point, however, is the relative absence (∼6%) of high-demand teacher assignments: assignments with scores that fell into the top third of the score scale. In fact, only 21 (<10%) of the 225 math teachers had even one assignment score in the high-demand category, and only two (<1%) teachers in the study had more than one assignment rated at that level.

#### 3.2.2 ELA

For ELA, there is a noticeable shift in the quality of student work in response to increased demand in the assignment, with a marked change in distribution as assignments move from low demand to medium and high demand. HLM analysis returned a standardized coefficient of 0.72 (*t* = 23.8) and an *R*^*2*^ value of 0.52, suggesting a strong relationship between ELA assignment demand and student work.

### 3.3 What is the relationship between assignment quality and other measures of teaching quality in math and ELA?

We analyzed the relationships between teacher assignment and student work scores and other measures used to characterize teaching quality. All correlations were run at the teacher level. Teacher assignment scores included only challenging classroom assignments since they were the only assignments that also included student work. All results described in the following subsections are presented in Table 7.

**Table 7:** Teacher-level correlations between challenging assignment quality, student work, and other measures of teaching quality.

Teacher Quality Measure	Math Assignment	Math Student Work	ELA Assignment	ELA Student Work
VAM	NS	0.15^∗^	NS	NS
MKT/Praxis^®^	NS	NS	0.22^∗∗^	0.28^∗∗^
CLASS™	NS	0.15^∗^	0.17^∗∗^	0.36^∗∗∗^
FFT (total)	NS	0.15^∗^	0.23^∗∗∗^	0.33^∗∗∗^
MQI (total)	NS	NS	–	–
PLATO (total)	–	–	0.13^∗^	0.24^∗∗^

*Note.* *p < 0.05. **p < 0.01. ***p < 0.001.

#### 3.3.1 Teacher knowledge

No statistically significant correlation was observed between quality of math assignments or student work and scores on the MKT teacher knowledge assessment. For ELA, positive and statistically significant correlations were observed for both assignments and student work.

#### 3.3.2 Observation protocols

For each observation protocol we report relationships with total observation scores. For math, the correlation of assignment scores with overall observation scores was not statistically significant for either protocol. However, small but statistically significant positive relationships with student work were observed for the domain-general protocols but not for the subject-specific MQI. For ELA, all three protocol total scores were statistically significantly and positively related to both teacher assignments and student work.

#### 3.3.3 VAM

No relationship was found between VAM and teacher assignment quality in math or ELA, nor is a relationship observed between ELA student work and VAM. For math, there is a statistically significant but small positive association between the quality of student work and VAM.

### 3.4 How are assignment scores related to contextual variables including teacher characteristics, class demographics, schools, or districts?

In this analysis, we attempt to understand contextual, assignment, and teacher factors associated with assignment quality. These analyses were conducted at the class section level to capture differences between two class sections that a teacher taught. Class section, teacher, and school variables were all incorporated as random effects to account for the nested structure of the data. In addition, a number of contextual variables were included in the model and treated as fixed effects. These variables were time of the school year (*season*) during which the assignment was given, assignment type, different scales, student grade (*grades 6–8*), and school district (*3 districts*).

The model also included predictor groups of variables intended to capture important contextual dimensions. A first group of predictors was considered *class section demographic variables* and included, for each section, percentage of students in each section identified as free/reduced-priced lunch eligible, minority, and English language learners. A second group of predictors was labeled as *prior achievement variables* and included mean class section prior achievement scores as well as the proportion of students in each class section classified as either gifted or special education. A third group of predictors was treated as *teacher quality variables* and consisted of teacher knowledge and VAM estimates.

To model the likelihood of different factors being associated with assignment ratings of different quality, scale scores were analyzed via a multi-level cumulative logit mixed model described in Appendix B. A full model incorporating all of the aforementioned contextual variables and predictors was fitted to the data to investigate their relationship with assignment ratings. Estimates from the full model are presented in Table 8 and elaborated on in the following subsections.

**Table 8:** Model estimates of contextual variables and assignment demand scores.

Contextual Variables^a^	Math	ELA
District A^b^	−0.06	0.12
District B^b^	0.89^∗∗∗^	−0.06
Season^c^	−0.14	−1.10^∗∗∗^
Assignment Type^d^	0.53^∗∗^	1.96^∗∗∗^
Grade 7^e^	−0.41^∗∗^	−0.07
Grade 8^e^	−0.42^∗∗^	0.41^∗^
Scale 2 f	−2.45^∗∗∗^	−1.51^∗∗∗^
Scale 3^f^	−2.72^∗∗∗^	−3.01^∗∗∗^

Teacher Quality Variables^g^	***p*** > **0.05**^h^	***p*** > **0.05**^h^
VAM	0.02	−0.15^∗^
MKT/Praxis	0.01	0.02

Class Section Demographic Variables^g^	***p*** < **0.01**^h^	***p*** < **0.01**^h^
Minority	−0.28^∗∗^	−0.16^∗∗^
FRLP	0.19^∗∗^	−0.13^∗∗^
ELL	−0.02^∗∗^	0.09^∗∗^

Prior Achievement Variables^g^	***p*** < **0.05**^h^	***p*** < **0.001**^h^
Gifted	0.03^∗^	0.10^∗∗∗^
Special Education	−0.02^∗^	0.02^∗∗∗^
Prior Test Scores	0.05^∗^	0.16^∗∗∗^

*Note.* *p < 0.05. **p < 0.01. ***p < 0.001. Coefficients are in logits.

a Describe the characteristics of the district, school, and classroom.

b Referenced against District C.

c Negative means Spring scores are lower.

d Positive means Challenging scores are higher.

e Referenced against Grade 6.

f Referenced against Scale 1.

g Represent blocks of variables that are evaluated via the likelihood ratio test.

h The *p* values in bold indicate whether each block represents a statistically significant improvement over the model including contextual variables only.

There was multicollinearity among the individual variables within each predictor group, making the estimates of single variables within each group problematic to interpret. Therefore, a series of likelihood ratio tests (LRTs) was conducted that evaluated the effect of including the group of variables in the model compared with a model that contained all variables but those belonging to the specific predictor group. The p-values of the LRT are presented in Table 8.

#### 3.4.1 Math

Statistically significant effects were observed for school district such that assignments from one district were more intellectually demanding than from the other two districts. Sixth-grade assignments were more intellectually demanding than assignments in older grades. Both of these findings suggest the possibility of curricular differences across grades and/or districts that might account for intellectual demand of assignments. As observed in other analysis, challenging assignments differed from typical assignments and scale distributions differed as well.

Class sections with larger proportions of minority, English language learner, and free or reduced price lunch students were associated with assignments that had lower intellectual demand. Class sections that higher prior achievement scores, more students classified as gifted, and fewer classified as special education had assignments with greater intellectual demand. However, there was not a significant difference in intellectual demand among classes that differed with respect to the teacher's knowledge and VAM scores.

#### 3.4.2 ELA

ELA relationships of contextual variables differed somewhat from math. No district effects were observed, and eighth grade had more challenging assignments than the lower grades. Additionally, there was an effect of season, such that assignments given in the second half of the school year were less challenging than those given in the first half. There were substantial differences across assignment type and scale.

Patterns among ELA predictor group variables paralleled math. Class sections with larger proportions of minority, English language learner, and free or reduced price lunch students were associated with assignments that had lower intellectual demand. Class sections that had students with higher prior achievement scores, more students classified as gifted, and fewer students classified as special education were given assignments with greater intellectual demand. However, there was not a significant difference in intellectual demand among classes that differed with respect to the teacher's knowledge and VAM scores.

## 4 Discussion

This study presents findings that describe how classroom artifacts can provide unique insights into the quality of instruction experienced by students in mathematics and ELA. These insights are complementary to those provided by existing measures, capturing both the instructional expectations set in a particular classroom and the student responses to these expectations. This research demonstrates that artifacts can provide explicit and reliably judged evidence about the intellectual demand of the work students are asked to do and the intellectual demand in the students’ response, shedding light on curricular and contextual factors as well as teaching quality. The study also provides evidence about the relationship between intellectual demand of assignments and other measures of teaching quality and contextual correlates.

A critical distinction in examining teaching has been the contrast of *teacher quality* with *teaching quality* (Kennedy, 2010). *Teacher quality* focuses on the individual and assumes a causal connection between what the teacher does and what the student learns. *Teaching quality* focuses on the teaching and the teacher as a central actor in a complex system that includes a host of contextual factors including curriculum, policy, and community (Bell et al., 2012). Studies of teaching quality more often include a focus on normative practice as a way of understanding consistencies in practice across a system. This research is an examination of teaching quality and avoids any claims of causal attribution to individual teachers. Instead, this study focuses on both differences and commonalities across classrooms as a way of understanding teaching quality situated in an understanding of the contextual factors. That is, artifacts provide evidence of instructional quality that is likely attributable to factors that include, but are not limited to, the teacher. This is a significant departure from teacher observations that tend to be centered on a teacher's strengths or weaknesses and, instead, focus on the actual intellectual demand asked of a student.

Evidence from assignments reinforces findings from observations that the intellectual demand of classroom interactions is very limited and relatively consistent across classrooms. As in observation studies, including the study from which these data are drawn (see Bell et al., 2014), dimensions associated with intellectual demand consistently score relatively low on different protocols (Gitomer et al., 2014) and are clustered around a narrow range at the lower part of respective scales. Score variance attributable to raters is very low, and scale inter-correlations are moderate. While the construct of intellectual demand is the basis of IDAP for both math and ELA, the specific features of design and performance that define the domain-specific IDAP are unique and consistent with the structure of particular disciplines. For both math and ELA, raters are able to reliably score artifacts and to differentiate scores on three scales designed to capture intellectual demand. In addition to this general pattern, there are specific results that are appropriate reported separately by domain.

The construct of intellectual demand is the basis of IDAP for both math and ELA. However, the specific features of design and performance that define the domain-specific protocols are unique and consistent with the structure of particular disciplines. For both math and ELA, raters are able to reliably score artifacts and to differentiate scores on three scales designed to capture intellectual demand. Therefore, the IDAP can be used effectively to rate artifacts as evidence of teaching quality.

In math, the intellectual demand of artifacts is quite low and narrowly distributed for all scales, regardless of whether the teacher categorized the artifact as typical or challenging. Though there is a statistically significant difference in scores for typical and challenging assignments, those that teachers select as more challenging show only very small increases in each of the scales. To the limited extent that math artifacts show more demand, students are more likely to produce work that reflects this demand. Of course, the demands expressed on an assignment do not always reflect the full expectations of the teacher—some may be communicated orally or through other habits developed in the classroom.

The lack of substantial variation in math assignment scores leads to two sets of findings addressing issues of teaching quality. First, correlations with other variables of interest are highly attenuated. Therefore, these correlations with other measures of teaching quality are either not significant or very small. However, the finding of district and grade effects suggests the potential of this kind of artifact analysis to identify systematic differences that are worthy of further exploration. For example, can curricular differences at either the district or grade level account for these differences? Also, score decreases were found to be attributable to reduced attention to real-world relevance in the higher grades.

From a teaching quality perspective, however, this lack of variation is, in fact, quite important. The consistent lack of intellectual demand in math assignments suggests that issues of teaching quality must be addressed systemically. Even the teachers with the highest artifact scores had artifacts that scored low relative to the IDAP scales. While there are significant contextual relationships with demographic and prior achievement variables, the overarching story is that even in classrooms characterized by favorable predictors, the intellectual demand of assignments is quite low. In terms of the protocol, across the vast majority of classrooms in the study, students are asked to solve routine math problems that require little more than procedural execution and lack extended communication or real-world connections. These insights are valuable to stakeholders in a multitude of ways. The most obvious would be to exert pressure on publishers to deliver materials that are relevant, rigorous, and constructive so that these tasks can then be implemented as such in the classroom. Additionally, the small distinction between typical and challenging can be interpreted as a potential area for professional development for teachers in terms of how to effectively provide challenge in the classroom. Finally, in these findings there is a clear call to researchers and educators to further explore the role of relevance in middle years education.

Findings in ELA classrooms are somewhat different. For ELA, many of the challenging assignments are qualitatively different from typical assignments. Challenging assignments, while still modest in terms of the overall scale, are more likely to ask students to provide elaborated communication, to engage in greater construction of knowledge, and to complete more authentic writing tasks in which they attempt to take on a role, even if at a relatively superficial level. The more intellectually demanding ELA assignments are strongly associated with student work that evidences greater intellectual demand.

ELA scores are positively correlated with observation scores. Classrooms with higher intellectual demand in assignments are also characterized by observation scores that describe stronger classroom interactions. These classrooms are also taught by teachers with higher teacher knowledge scores. There are also significant contextual relationships with demographic and prior achievement variables.

For ELA only, there is an effect of the season during which the assignment is given. Assignments given in the spring are less demanding than those given in the fall. One conjecture is that the decrease may be reflective of an increased focus on standardized test preparation in the spring. Another is that teachers may experience planning fatigue as the year progresses. Also, assignments in eighth grade were more challenging than those in sixth grade, although no difference was noted for seventh grade. Further study is needed to understand this trend and whether it may be related to a perceived need that deeper tasks are required in preparation for high school.

Artifact research does allow for some important insights that are not always picked up by other measures. For example, the differences between the intellectual demand asked of students from different subgroups would be critical to key stakeholders in addressing equity of access. Additionally, if it is found that the spring drop in demand is related to the current timeframe of high-stakes testing, a response may be needed in either revising the testing in terms of intent and composition of the tests themselves.

Across both domains, correlations of assignment scores with VAM are not statistically significant, and this finding raises issues deserving of further study and adds to the argument for the drop in demand in preparation for the standardized testing, from which VAM is calculated, in the spring. It may be that the items on achievement tests that are used to determine VAM estimates are not asking students to engage in intellectually demanding tasks as valued by the IDAP. Similarly, it may be that improvement in achievement test scores can be made by simply focusing on the improvement of skills associated with low-demand tasks. A much better understanding of the alignment of test demands with assignment demands is needed to better interpret this lack of consistent relationship between measures.

Grade, district, and seasonal effects suggest the possibility that assignment differences reflect differences in mandated curriculum in addition to any differences that can be attributed to individual teachers. Current teacher evaluation efforts have placed a very substantial focus on individual teacher differences, but there are likely broader contextual factors such as curriculum that also need to be given attention (Gitomer, 2017). The link between mandated curriculum reform and teacher interpretation, as explored by Cohen and Hill (2000), merits further investigation.

### 4.1 Limitations

It is important to recognize features of the research that have implications for interpreting the study's findings. As noted, the Newmann et al. (2001) framework represents a particular perspective on instruction. Different frameworks might support different inferences about the quality of assignments. Nor does the framework used in this study consider important teaching and learning issues such as learning progressions, adaptive materials for students with special needs, culturally responsive pedagogy, and the relationship of artifacts to curriculum design. The IDAP may have more or less utility in studying these kinds of problems.

The issue of teachers selecting challenging and typical assignments raises potentially interesting questions as well. In this study, teachers were given limited guidance in deciding what constituted a challenging assignment. Thus, inferences in this study are not simply about the quality of an assignment but also include teacher choice of the assignment. While the student data suggests that the teachers were differentiating assignments in ways that were sensitive to the protocol, there is a question of whether different, and perhaps more directive, sampling procedures would lead to different interpretations.

Finally, as with most artifact studies, judgments are made on the basis of evidence contained in the artifact documents. Evidence of what transpired in the classroom around the enactment of the artifacts is not available. Certainly interactions between teachers and students might strengthen or weaken the expectations that can be inferred by only considering the physical documentation.

### 4.2 Conclusions

As a measure of teaching quality, assignments have the potential to contribute to an understanding of the extent to which intellectual demand is present in classrooms and across classrooms within schools and districts. These insights can then be used to address development needs in each of these areas leading to improvement in teaching quality and ultimately to improvement of student outcomes (Yoon, Duncan, Lee, Scarloss, & Shapley, 2007). By examining score patterns as we have done, schools and districts may be able to better identify both the extent to which students are asked to engage in intellectually meaningful work as well as the teacher, classroom, and institutional factors that contribute to students’ learning experience. Further, with this insight, schools and districts could determine priorities for improvement at a local or system level.

## Funding

This work was supported by the Bill & Melinda Gates Foundation, Grant# OPP52048, as part of the Understanding Teaching Quality (UTQ) project.

## Appendix A

### IDAP protocol

#### Math - Teacher assignment scoring

##### Scale 1 - (Written mathematical communication)

**3** = *Analysis/Persuasion/Theory*. The task requires the student to show his/her solution path and to explain the solution path with evidence. **2** = *Report/Summary*. The task requires the student to show her/his solution path or requires explanation of why. **1** = *Just answers*. The task requires little more than answers. Students may be asked to show some work.

##### Scale 2 - (Conceptual Understanding)

**4** = The dominant expectation of the assignment requires substantial demonstration of conceptual understanding that is related to one or more mathematical ideas. **3** = The dominant expectation of the assignment requires some demonstration of conceptual understanding that is related to one or more mathematical ideas. **2** = The dominant expectation of the assignment relates to a mathematical idea but primarily requires demonstration of procedural knowledge. **1** = The mathematical expectation of the assignment relates only tangentially, if at all, to a mathematical idea.

##### Scale 3 - (Real-world connection)

**4** = The assignment clearly addresses an authentic situation that entails relevant mathematical questions, issues, or problems that are encountered in the real world and specifies that the work will be meeting the needs of a real and appropriate audience, one other than a teacher or student peers. **3** = The assignment clearly addresses an authentic situation that entails relevant mathematical questions, issues, or problems that are encountered in the real world but does not specify that the work will be meeting the needs of a real and appropriate audience. **2** = The assignment makes an attempt to address mathematical questions, issues, or problems that are encountered in the real world, but the connection is superficial, tangential, contrived, or weak. **1** = The assignment makes a minimal attempt to address the mathematical questions, issues, or problems that are encountered in the real world.

#### Math - Student work

##### Scale 1 - (Written mathematical communication)

**3** = Student work demonstrates a complete indication of the solution path with complete and appropriate explanation/justification OR complete and appropriate explanation/justification of the student's thinking and conclusions. **2** = Student work demonstrates a clear indication of the solution path but little or no appropriate explanation or justification. **1** = Student work demonstrates little more than an answer, with little or no indication of the solution path and little or no appropriate explanation or justification.

##### Scale 2 - (Conceptual understanding)

**4** = Student work demonstrates substantial conceptual understanding of important mathematical ideas and is free of misconceptions. **3** = Student work demonstrates some conceptual understanding of important mathematical ideas and may contain minor misconceptions. **2** = Student work demonstrates partial conceptual understanding of important mathematical ideas and may contain major misconceptions. **1** = Student work demonstrates little or no conceptual understanding of mathematical ideas.

##### Scale 3 - (Reasoning)

**4** = Student work demonstrates appropriate and successful use of reasoning. **3** = Student work demonstrates reasoning, but it is not entirely appropriate or successful. **2** = Student work demonstrates some use of reasoning strategies. **1** = Student work represents primarily the reproduction of fragments of knowledge OR the application of previously learned procedures OR completely inappropriate reasoning.

#### ELA - Teacher assignments

##### Scale 1 - (Elaborated communication: expository//narrative)

**4** = The task requires extended writing and asks students to make an assertion—stating a claim OR drawing a conclusion OR suggesting a generalization—AND to support the assertion with evidence offered by examples, details, illustrations, facts, AND/OR reasons.//The task requires extended writing and calls for imaginative texts that demonstrate the tone, style, conventions, and content of the genre AND that include detail. **3** = The task requires extended writing and asks students to EITHER make an assertion—stating a claim OR drawing a conclusion OR suggesting a generalization—OR to support a given assertion with evidence offered by examples, details, illustrations, facts, AND/OR reasons.//The task requires extended writing and calls for imaginative texts, BUT it does not require demonstration of the characteristics of the genre. **2** = Short answers are sufficient to complete the task; the task requires only one or two sentences, clauses, or phrases that complete a thought. **1** = The task is a fill-in-the-blank or multiple-choice task.

##### Scale 2 - (Construction of knowledge)

**3** = The dominant expectation of the task is that students construct knowledge and/or an idea—to generate and explore ideas through interpretation, analysis, synthesis, and/or evaluation of information or through the use of literary strategies and techniques. **2** = There is some expectation that students construct knowledge or an idea—to generate and explore ideas through interpretation, analysis, synthesis, and/or evaluation of information or through the use of literary strategies and techniques for part of the task, BUT these skills are not the dominant expectation of the task. **1** = There is very little or no expectation for students to construct knowledge or an idea. Students can satisfy all or almost all of the requirements of the task by reproducing information they have read, heard, or viewed. For imaginative tasks, students are given very little or no direction in the use of literary strategies and techniques in order to construct an idea.

##### Scale 3 - (Real-world connection)

**4** = The task requires students to take on a real-world role. The task also calls for a product that achieves a purpose beyond the simple demonstration of academic competence. In addition, the task requires that work be submitted to an audience other than teachers or students as graders. **3** = The task requires students to take on a real-world role. The task also calls for a product that achieves a purpose beyond the simple demonstration of academic competence. There is no requirement that the work be submitted to a real audience. **2** = The task requires students to take on a role but one that students could not realistically assume now or in the future. The primary purpose of the task is to demonstrate academic competence to teachers or students as graders. **1** = The task does not specify a role for the student. The primary purpose for the task is to demonstrate academic competence.

#### ELA - Student work

##### Scale 1 - (Elaborated communication: expository//narrative)

**4** = The work includes extended writing that makes an assertion AND supports it with relevant evidence. The work ALSO is sufficiently developed, internally coherent, and effectively organized.//The work includes extended writing and detail. The work ALSO demonstrates the tone, style, conventions, and content of the genre AND evokes meaning through these literary devices. **3** = The work includes extended writing that makes an assertion AND supports it with relevant evidence. However, the work lacks sufficient development, internal coherence, and/or effective organization.//The work includes extended writing and detail. The work ALSO demonstrates the tone, style, conventions, and content of the genre. **2** = The work includes extended writing that EITHER makes an assertion OR provides evidence for a given assertion but does not do both.//The writing does not demonstrate the characteristics of the genre well, although it offers more sustained writing than isolated sentences or short answers. **1** = The writing is a series of isolated sentences or short answers that do not support a central idea, or the writing responds to fill-in-the-blank or multiple-choice tasks.

##### Scale 2 - (Construction of knowledge)

**3** = The dominant part of the work constructs knowledge or an idea; the work generates and explores ideas through interpretation, analysis, synthesis, and/or evaluation of information or through the use of literary strategies and techniques. **2** = The work demonstrates moderate construction of knowledge or an idea. A portion of the work generates and explores ideas through interpretation, analysis, synthesis, and/or evaluation of information or through the use of literary strategies and techniques, BUT these skills are not the dominant part of the work. **1** = There is very little or no construction of knowledge or an idea. All or almost all of the work could have been generated by reproducing information the student has read, heard, or viewed. For imaginative tasks, literary strategies and techniques are not used to construct an idea.

##### Scale 3 - (Language conventions)

**4** = The writing demonstrates competent command of spelling, vocabulary, grammar, and punctuation, AND effective use of language resources. The writing is almost error free. **3** = The writing demonstrates competent command of spelling, vocabulary, grammar, and punctuation. There may be some errors, but they present no problem for understanding the student's meaning. **2** = There are frequent errors in spelling, vocabulary, grammar, and punctuation, and they may interfere with the student's meaning, making the work difficult to understand. **1** = The writing is a series of isolated sentences or short answers that do not support a central idea, or the writing responds to fill-in-the-blank or multiple-choice tasks, or the writing is not the student's own.

## Appendix B

### Cumulative logit mixed model

The model was coded using the clmm function (with default settings) in the R package ordinal (Christensen, 2015). The dependent variable was the raw scale rating (ordinal) and the predictors in the different models as described in the manuscript. This is essentially an extension of the well-known multilevel/hierarchical linear models (Raudenbush & Bryk, 2002) for ordinal dependent variables. Fitting the model with ordinal variables requires using a logit link, and thus the estimated coefficients are in the logit-link as well. The hierarchical nature of the data can be accommodated with the software estimating both the fixed and random effects.

## Footnotes

1 One classroom was team-taught resulting in an extra set of assignments.

2 The total scores were modeled as follows: $\log\left(\frac{P_{nmijk}}{P_{nmij\left(k-1\right)}}\right)=B_{n}-D_{i}-C_{j}-\;F_{k}$, where $B_{n}$ is the overall quality of the assignment, $D_{i}$ is the difficulty of the particular scale *i*, $C_{j}$ is the severity of the rater *j*, $F_{k}$ is the threshold difficulty of scale rating k vs k-1, and $P_{nmijk}$ is the probability of receiving rating k, and $P_{nmij\left(k-1\right)}$ is the probability of receiving rating k-1.
